# Editorial: Metal-free oxidative transformations in organic synthesis

**DOI:** 10.3389/fchem.2022.956779

**Published:** 2022-08-08

**Authors:** Fateh Veer Singh, Toshifumi Dohi, Ravi Kumar

**Affiliations:** ^1^ Chemistry Division, School of Advanced Sciences, Vellore Institute of Technology (VIT), Chennai Campus, Chennai, Tamil Nadu, India; ^2^ College of Pharmaceutical Sciences, Ritsumeikan University, Kusatsu, Shiga, Japan; ^3^ Department of Chemistry, J.C. Bose University of Science and Technology, YMCA, Faridabad, Haryana, India

**Keywords:** metal-free, sustainable approach, oxidation, green methods, organocatalysts

## Introduction

The tools of synthetic approaches must be continuously expanded and enriched to facilitate the sustainable production of chemicals. The development of processes with considerably superior environmental and industry-relevant credentials is one of the prime objectives for organic chemists. Most of the organic transformations, including C-C and C-X bond-forming cross-coupling reactions, and cross dehydrogenative-coupling reactions generally rely on the prerequisite of transition-metal catalysts and harmful organic solvents. Hence, there is a dire need of developing green synthetic strategies by avoiding the use of transition-metal catalysts and hazardous organic solvents. Metal-free oxidative transformations have emerged as an important alternative to metal catalysis in the past few decades. Even, in some cases, metal-free catalysis has more advantages over metal-catalyzed reactions, such as exceptional performance, better selectivity, recyclability, and higher substrate tolerance. This thematic issue includes research papers covering key aspects of metal-free transformations.

Furfural and its derivatives are an important class of biomass platform compounds and, have been widely used in industries. Reported synthetic methods for these compounds involve the use of inorganic acid catalysts (Zhou et al., 2021), such as sulfuric acid, hydrochloric acid, phosphoric acid (Yemis and Mazza, 2011), and also the solid acid [Nb_2_O_5_-MCM-41] (Garcia-Sancho et al., 2013). However, these methods suffer many disadvantages. In the case of sulfuric acid, hydrochloric acid, and phosphoric acid, the yield of the furfural derivatives was only upto 37.5% while the synthetic method using [Nb_2_O_5_-MCM-41] was suffering from less selectivity and high cost. These limitations were overcome by the use of ionic liquids (ILs), in particular, the ionic liquids bearing diimidazole rings (Ni et al., 2017; Liu et al., 2019) that give superior results because of their better hydrophobic character and good thermal stability. Xiong and coworkers explored the catalytic utilization of various diimidazole hexafluorophosphates, with varying carbon chain lengths, under microwave irradiation for the conversion of xylose into furfural. Salient features of the developed protocol are the shorter reaction time, 1/40th of the reaction time required for the conventional method, with a slight increase in the yield. Further, the catalytic activity of these catalysts was available after being recycled five times.

Hypervalent iodine compounds were discovered a long time ago and, in 1886 a German chemist, C. Willgerodt synthesized (dichloroiodo)benzene as the first stable polyvalent organic iodine compound (Willgerodt, 1886). This achievement was quickly followed by the preparation of many other iodine compounds, including the most common reagents (diacetoxyiodo)benzene and iodosylbenzene (Willgerodt, 1892), 2-iodoxybenzoic acid (IBX) (Hartmann and Meyer, 1893), and the first examples of diaryliodonium salts reported by Hartmann and Meyer (1894). Over the last few decades, hypervalent iodine chemistry has evolved from being a mere curiosity to a most prosperous field in organic synthesis (Wirth, 2016; Yoshimura and Zhdankin, 2016; Parra, 2019; Dohi, Han and Kumar, 2021; Singh and Wirth, 2021; Kumar et al., 2022; Rimi et al., 2022). The most important representative of pentavalent iodine compounds is IBX, apart from Dess-Martin Periodinane (DMP) (Hartmann and Meyer, 1893). Despite having shortcomings such as explosive nature and insolubility in common organic solvents, IBX is widely applied for organic oxidative reactions. Nageswar et al. presented the recent developments related to the IBX-mediated organic transformations in heterocyclic chemistry, in particular, from 2010 onwards.

With progress in active pharmaceutical ingredients (API) and functional materials, the demand for manufacturing processes utilizing resource-recyclable reagents and highly atom-economical synthetic methods has enhanced substantially (Horvάth and Anastas, 2007; Sheldon, 2012; Hayashi, 2016; Horvάth, 2018). Yamamoto et al. developed a metal-free organocatalytic practical approach for the synthesis of diverse nitrogen heterocycles. Salicyclic acid and its derivatives were efficiently used as organocatalysts under atmospheric oxygen. Synthesis of the quinazolines was successfully achieved by the group with excellent atom economy, an environmental factor (E-factor) of 2.7, and reaction mass efficiency (RME) of 73%. In addition, the synthesis of quinazolines was scaled up to 10 mmol.

Indole, as well as quinoline, is widely present in natural products and medicines and, their functionalization gives a wide array of essential targets. However, the C-3 modification of these rings is quite challenging and, has been the field of extensive research. A one-step metal-free C-3 functionalization of the quinoline ring leading to C-C bond formation is recently reported in the literature (Sangher et al., 2020). Taking into consideration the concepts of green chemistry, a mild C-H functionalization approach for the construction of diverse indole-containing heterocyclic frameworks was established by Chen et al. The one-pot three-component coupling of α-Amino aryl ketones, indoles, and perbromomethane afforded biologically significant 2-(1-bromo-1H-indol-3-yl)-2-imino-carbonyls involving the simultaneous construction of three different new bonds (C=N, C–C, and N-Br). Salient features of the methodology involve mild reaction conditions, atom economy, and transition metal-as well as oxidant-free approach.

## Perspectives

This Research Topic of articles showcases different aspects of metal-free approaches toward achieving various valuable organic transformations. Irrespective of the fact that there are continuous expansions in this field, there are still many areas yet to be explored. Refereeing the advantages of metal-free oxidative transformations, it seems certain that these reactions will be further utilized in the development of sustainable synthetic methods.
